# The PAGODAS protocol: pediatric assessment group of dengue and *Aedes* saliva protocol to investigate vector-borne determinants of *Aedes*-transmitted arboviral infections in Cambodia

**DOI:** 10.1186/s13071-018-3224-7

**Published:** 2018-12-20

**Authors:** Jessica E. Manning, Fabiano Oliveira, Daniel M. Parker, Chanaki Amaratunga, Dara Kong, Somnang Man, Sokunthea Sreng, Sreyngim Lay, Kimsour Nang, Soun Kimsan, Ly Sokha, Shaden Kamhawi, Michael P. Fay, Seila Suon, Parker Ruhl, Hans Ackerman, Rekol Huy, Thomas E. Wellems, Jesus G. Valenzuela, Rithea Leang

**Affiliations:** 1 Laboratory of Malaria and Vector Research, National Institute of Allergy and Infectious Diseases, National Institutes of Health, Rockville, Maryland USA; 2 Department of Population Health and Disease Prevention, University of California, Irvine, California, USA; 3 National Center of Parasitology, Entomology, and Malaria Control, Phnom Penh, Cambodia; 4 Biostatistics Branch, National Institute of Allergy and Infectious Diseases, National Institutes of Health, Bethesda, Maryland USA

**Keywords:** Arbovirus, Vector, Saliva, Vaccines, Mosquito, Cohort, Seroprevalence

## Abstract

**Background:**

Mosquito-borne arboviruses, like dengue virus, continue to cause significant global morbidity and mortality, particularly in Southeast Asia. When the infectious mosquitoes probe into human skin for a blood meal, they deposit saliva containing a myriad of pharmacologically active compounds, some of which alter the immune response and influence host receptivity to infection, and consequently, the establishment of the virus. Previous reports have highlighted the complexity of mosquito vector-derived factors and immunity in the success of infection. Cumulative evidence from animal models and limited data from humans have identified various vector-derived components, including salivary components, that are co-delivered with the pathogen and play an important role in the dissemination of infection. Much about the roles and effects of these vector-derived factors remain to be discovered.

**Methods/Design:**

We describe a longitudinal, pagoda (community)-based pediatric cohort study to evaluate the burden of dengue virus infection and document the immune responses to salivary proteins of *Aedes aegypti*, the mosquito vector of dengue, Zika, and chikungunya viruses. The study includes community-based seroprevalence assessments in the peri-urban town of Chbar Mon in Kampong Speu Province, Cambodia. The study aims to recruit 771 children between the ages of 2 and 9 years for a three year period of longitudinal follow-up, including twice per year (rainy and dry season) serosurveillance for dengue seroconversion and *Ae. aegypti* salivary gland homogenate antibody intensity determinations by ELISA assays. Diagnostic tests for acute dengue, Zika and chikungunya viral infections will be performed by RT-PCR.

**Discussion:**

This study will serve as a foundation for further understanding of mosquito saliva immunity and its impact on *Aedes*-transmitted arboviral diseases endemic to Cambodia.

**Trial registration:**

NCT03534245 registered on 23 May 2018.

## Background

Recently, important *Aedes* mosquito-borne viruses such as chikungunya (CHIKV), yellow fever, dengue (DENV), and Zika (ZIKV) have re-emerged and caused public health alarm on a global scale [1–3]. Accumulating evidence in animal models suggest that mosquito saliva proteins (MSPs) increase the infectivity of these arboviruses [4–10]. Despite the evidence that mosquito saliva overall facilitates infection, relatively few proteins have been identified as immunomodulatory. *Aedes aegypti* saliva has over 20 unique abundant secreted proteins, most of unknown function [11]. Further complicating our understanding, arthropod salivary components are highly diverse with some proteins facilitating infection, while others may impede viral infection when tested in isolation [12–14]. Moreover, viral infection and dissemination differs between needle-inoculated *versus* arthropod-delivered infection models in mice, hamsters, beagles and primates [6, 7, 15, 16].

The relationship between mosquito saliva reactivity and clinical disease thus needs further investigation. An *Aedes aegypti* N-terminus 34-kDa salivary peptide of unknown function is a known marker of *Aedes* spp. mosquito exposure in humans, but depends on the location and timing of the exposure [17]. Less information is available on how salivary proteins may impact risk of arboviral infection in humans. Indeed, the few human studies devoted to disease development have been limited by power, retrospective design, or convenience sampling [18, 19]. To address some of the key knowledge gaps, we have established a protocol to study dengue seroprevalence and *Aedes aegypti* saliva reactivity *via* a prospective cohort of at-risk children in a country endemic to multiple *Aedes*-transmitted diseases.

Cambodia, a country with an estimated population of approximately 16 million, has multiple mosquito-borne diseases caused by various malaria parasites and DENV, CHIKV, ZIKV, and Japanese encephalitis virus (JEV) [20]. The risk of arboviral disease may be increasing in the next decade as the Cambodian urban population is expected to double before 2030 [21]. Rapid urbanization, congestion, land-use change and flooding may further contribute to increased risk in mosquito or vector-borne diseases in this region [22].

With regard to *Aedes*-transmitted infections, DENV is most common in Cambodia and is highly endemic with year-round transmission, peaking from July to November when rainfall is at its highest. Transmission is highly focal but is found in both rural and urban areas in Cambodia [23, 24]. The National Dengue Control Programme (NDCP) has a pediatric hospital-based syndromic surveillance system across the country. At five sentinel sites, clinically suspected cases are sent to Pasteur Institute to confirm DENV infection *via* RT-PCR detection of the virus [20]. Epidemiological studies otherwise rely upon reports of clinically diagnosed dengue-like cases (occasionally confirmed by SD Bioline Dengue Duo rapid tests when available) from hospitalized children under 16 years of age, with little assessment or data in older adults who are less likely to seek care. In Cambodia, average DENV incidence appears to be highest in those under 7 years of age (approximately 41 cases per 1000 person-seasons in one active community-based surveillance study from 2006 to 2008) [24]. Given its reliance on a clinical reporting system, national DENV incidence data likely underestimates true DENV incidence in Cambodia by 3 to 27-fold, per capture-recapture analyses [25]. Among other arboviruses, CHIKV outbreaks are occasionally documented in Cambodia, e.g. one village in Kampong Speu Province experienced a 44% attack rate in 2012 [26]. ZIKV transmission is uncharacterized and may sporadically occur in Cambodia [20].

Considering the vector abundance of *Aedes* mosquitos and the presence of multiple *Aedes*-transmitted viruses in Cambodia, we chose to establish a prospective pediatric cohort using local pagodas (religious temples) for community recruitment and follow-up. This study protocol aims to define the seroprevalence of antibodies to both DENV and *Aedes aegypti* salivary gland homogenate (SGH), describe the epidemiology of symptomatic *Aedes*-transmitted infection in children in this community, characterize the demographic and entomological factors that contribute to DENV infection, and establish the magnitude of human antibody response to *Ae. aegypti* saliva. These data will establish the groundwork on which to better understand the roles of vector-borne determinants of arboviral infection.

## Methods/Design

### Study design and objectives

This is a prospective pagoda (community)-based cohort study with the following primary objectives: (i) to determine the seroprevalence of antibodies indicating previous *Aedes*-transmitted DENV infection in children; and (ii) to describe the seropositivity to *Aedes* salivary gland homogenate (Table 1). The study will screen up to 1200 and recruit up to 771 children aged 2–9 years for study enrollment and then follow them longitudinally for three years. The study is being conducted over three years given the high rate of variability in *Aedes*-transmitted disease transmission from year to year. The study is focused on children aged 2–9 years of age as they have the greatest burden of disease in Cambodia and thus offers comparability to other pediatric DENV cohorts [23–25, 27].

**Table 1:** Study objectives and endpoints

Objectives	Endpoints	Justification for endpoints
• Primary		
Describe the seroprevalence of dengue infection (both symptomatic and inapparent) in children aged 2–9 years-old in Kampong Speu	Prevalence of symptomatic and inapparent dengue infection (serotypes 1–4) as detected semiannually *via* ELISA (binary outcome present/absent) over a 3-year period in Kampong Speu in children aged 2–9 years-old	Detailed knowledge of dengue seroprevalence and transmission season variability will help establish an epidemiological foundation to prepare for larger future studies such as disease incidence studies or vector interventional trials
Describe the seroprevalence of reactivity to *Ae. aegypti* salivary gland proteins in children aged 2–9 years-old in Kampong Speu	Prevalence of *Ae. aegypti* salivary gland homogenate reactivity as detected by ELISA assay (binary outcome present/absent) during wet and dry seasons over a 3-year period in Kampong Speu in children aged 2–9 years-old	Characterizing the *Ae. aegypti* salivary protein reactivity profile in Cambodians is the first step prior to assessing how *Ae. aegypti* saliva exposure modulates disease in humans
• Secondary		
Describe epidemiology of symptomatic *Aedes*-transmitted infections in a Cambodian pediatric population	Positive RT-PCR result for diagnosis of dengue, chikungunya, and Zika viruses (or IgM capture ELISAs for dengue as needed)	Dengue is the predominant flavivirus, but other circulating arboviruses complicate the serologic diagnosis of prior immunity and it will be important to characterize this burden given the ease and little cost added to run the multiplex RT-PCR compared to a DENV RT-PCR alone
Establish and characterize spatial relationships between population abundance of *Ae. aegypti* (through entomological indicators), magnitude of human antibody response to *Ae. aegypti* mosquito saliva, and disease development	Geographical information system with all data components (mosquito catch sites, houses, schools) referenced by latitude and longitude in addition to a series of map layers (point maps, smoothed maps) to evaluate relationships between IgG intensity of human antibody response (ΔOD per ELISA) to *Ae. aegypti* salivary homogenate, entomological indices (e.g. House index defined as larvae present per 100 houses surveyed), and disease development	These data and analyses will allow for detailed spatio-temporal analysis of the data at individual, house and community levels. Similar published methods (except different entomological indices) were used for assessment of *Anopheles* salivary protein exposure at the Thai-Burma border for risk of malaria transmission [53]
Characterize temporal dynamics of human antibody reactivity to *Ae. aegypti* salivary gland homogenate	Seroconversion to *Ae. aegypti* salivary homogenate in relationship to season (wet *versus* dry) and collected time-dependent variables defined as mean and maximum rainfall, temperature and humidity	The natural time course of salivary protein exposure, particularly in confirmed arboviral disease settings, has not been well characterized. Retrospective repeat analyses of non-diseased samples have suggested that salivary IgG antibodies may last 30–40 days or possibly up to 4 months [18]. Seasonal variation and climate factors are implicated in both disease transmission and vector abundance [54, 55]. Quantifying vector-host interaction at the individual level in the context of more global factors is necessary to further understanding saliva immunity, disease transmission, and outbreak model prediction
Identify specific immunodominant molecules in *Ae. aegypti* saliva for functional characterization	Western blot analysis of sera from participants with strongest ELISA positivity to *Ae. aegypti* whole salivary gland homogenate compared to *Anopheles* and *Culex* to assess cross-reactive immunogenicity to mosquito saliva *versus* specific *Aedes* markers	This next layer of analysis to identify specific immune-dominant molecules, from a whole-protein approach, will provide the highest probability of identifying novel proteins, irrespective of immune-modulatory function, that can then be characterized. It is essential to assess reactivity to the saliva of *Anopheles* and *Culex* in order to exclude cross-reactive antigens and select for *Aedes*-specific markers
Characterize wild-caught *Aedes* sialotranscriptomes (transcriptomes of mosquito salivary glands) and midgut composition	Capture a minimum of 25 female *Ae. aegypti* mosquitos for transcriptional comparison to LMVR-reared *Ae. aegypti* mosquitos	Wild-caught *Ae. aegypti* may harbor considerable difference to inbred mosquito strains maintained in insectaries. Differences in salivary and midgut composition may help explain unique vector competence aspects and immunogenicity of the strains of *Ae. aegypti* in Cambodia
• Tertiary/Exploratory		
Investigate a relationship between the magnitude of human antibody response to *Aedes* salivary gland homogenate to subsequent disease development to *Aedes*-transmitted disease	Correlate the incidence of *Aedes*-transmitted arboviral infection (as defined below) with seroconversion to *Ae. aegypti* salivary homogenate	Retrospective human studies have identified seropositivity to *Ae. aegypti* proteins proving that humans mount antibody responses to specific *Ae. aegypti* salivary proteins [17–19, 40]. Mice studies demonstrate *Ae. aegypti* salivary protein immunization as protective of disease whereas co-inoculation of virus with saliva worsens disease
Characterize the presence and types of alpha-thalassemia variants in the Cambodian population	Presence or absence of alpha thalassemia as determined by genotyping of alpha globin deletions and variants in cohort participants and targeted sequencing of these candidate genes	Dengue-infected patients with alpha thalassemia clinically present differently than patients without erythrocyte variants [49, 50]. In *vitro* studies have also shown that cells derived from thalassemia trait carriers have reduced susceptibility to DENV infection. Describing the presence of alpha-thalassemia in the Cambodian population as well as the associated alpha globin deletions will be important for future studies to investigate the link between alpha thalassemia and arboviral disease development
Characterize pattern of *Aedes* salivary reactivity and cellular immune responses to saliva across baseline, final, acute and convalescent time points of *Aedes*-transmitted disease	Evaluation of the cellular immune response to *Aedes* whole salivary gland homogenate (e.g. assess T-cell activation or cytokine production by vector saliva) in participants at baseline, final study visit, and time points after with RT-PCR-confirmed infection with DENV, CHIKV, or ZIKV	Understanding cellular immunity to *Aedes* saliva will inform vaccine design. An ultimate aim of identifying a small peptide antigen in *Aedes* saliva would be its use as an adjuvant in conjunction with an arboviral-specific antigen for vaccine development

Twice per year, during dry (March) and wet (August) seasons, participants come to their local pagoda, a community temple, to provide blood samples for tests of seroconversion after DENV infection and to characterize their *Aedes* salivary protein seroconversion profiles (Table 2). At any time of year, febrile children are referred to the district hospital where putative cases of febrile *Aedes*-transmitted arbovirus infections can be identified by symptoms (e.g. fever, myalgias, headache, rash). For febrile cases with positive rapid diagnostic tests or for those with a high index of clinical suspicion, confirmatory tests of viral shedding of DENV, CHIKV or ZIKV virus are performed *via* RT-PCR.

**Table 2:** Schedule of activities

Procedure	Screen and enroll	Dry ± 28 days	Wet ± 28 days	Dry ± 28 days	Wet ± 28 days	Dry ± 28 days	Wet ± 28 days	Febrile sick visit ± 2 days	Convalescent visit Day 7 ± 2 days	Convalescent visit Day 28 ± 4 days
Informed consent	×									
Demographics	×									
Baseline medical history questionnaire	×									
Baseline physical exam	×									
Interval exam and questionnaire		×	×	×	×	×	×			
Concomitant medication review	×	×	×	×	×	×	×	×	×	×
Sick visit exam (with height and weight) and questionnaire								×		
Convalescent exam and questionnaire									×	×
Height and weight	×						×	×		
Vital signs	×	×	×	×	×	×	×	×	×	×
Dengue rapid diagnostic test (RDT)								×		
RT-PCR multiplex for acute arboviral diagnosis								× (if RDT+)		
Sera collection for ELISAs	×	×	×	×	×	×	×	× (if RDT+)	×	
Hemoglobin		×								
Alpha-thalassemia screening		×								
Peripheral blood mononuclear cells	×						×	× (if RDT+)		×
Complete case report forms	×	×	×	×	×	×	×	×	×	×

*Note*: Dry seasons visits take place every March whereas wet season visit take place every August

To reduce bias from loss to follow-up, the study team enrolls volunteers who are located within 5.5 km of the district hospital, will do follow-up sampling in the community, and will provide incentives commensurate with the previous studies approved by the Cambodian ethics committee.

### Study population and site

**Fig. 1:**
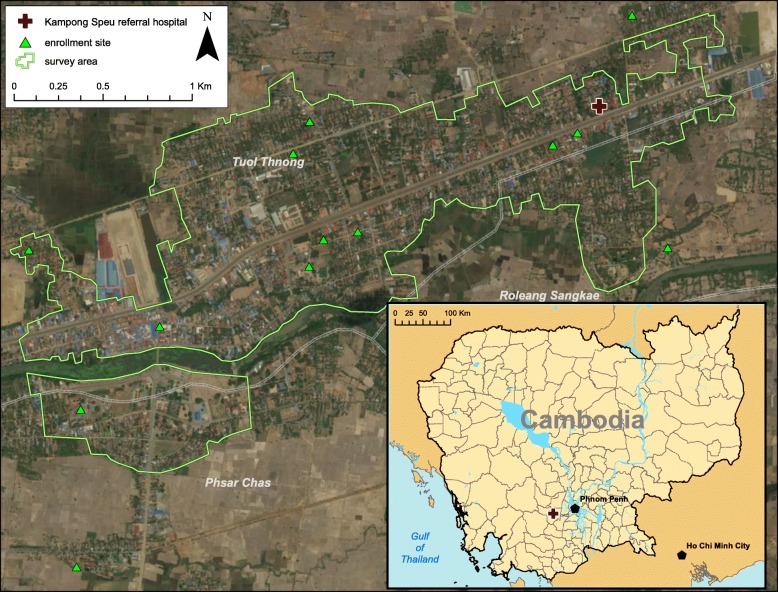
Primary study area. The inset map (bottom right) indicates the location of Chbar Mon town, Kampong Speu (red cross) in Cambodia. The main map indicates the study area (with a green outline) and recruitment sites (green triangles) where participants are recruited for immunological indicators of mosquito exposure

The study is carried out in Chbar Mon town, a peri-urban area with an estimated population approximately 60,000 located 44 km west of Phnom Penh city center (Fig. 1). Over 2000 primary school students who reside in 4 km^2^ area of the three easternmost and populous communes. This area includes a comparable but unconfirmed number of children aged 0–6 years old who have yet to enter school. Inclusion and exclusion criteria are listed in Table 3.

**Table 3:** Study eligibility criteria

Inclusion criteria	Exclusion criteria
(i) Provision of signed and dated informed consent form	(i) Current or prior use of immunosuppression in the previous six months
(ii) Stated willingness to comply with all study procedures and availability for the duration of the study	(ii) Treatment with an investigational drug or vaccine in previous six months
(iii) Male or female, aged 2–9 years	(iii) Any event or information deemed unsafe at the discretion of the principal investigator
(iv) Live within approximately 5.5 km of the Kampong Speu Referral hospital;	
(v) In good general health evidenced by medical history	
(vi) Willing to allow biological samples to be stored for future research	

National Dengue Control Programme data indicate that Kampong Speu is one of the higher burden provinces with a cumulative incidence of approximately 50 per 100,000 persons (over last three years based on 2013 census data). This number was similar to the national programme’s estimation of cumulative incidence in Kampong Cham, where rates of laboratory-confirmed cases ranged from 25 to 100 per 100,000 persons in 2015–2017 (unpublished national programme data).

### Community engagement

Engaging community stakeholders is critical to the success of a community-based cohort in Cambodia and is the basis of the effective collaboration between the National Dengue Control Programme, the provincial health departments, and the National Institute of Allergy and Infectious Diseases (NIAID) in conjunction with the Cambodian National Center for Parasitology, Entomology, and Malaria and Control (CNM). Chbar Mon is the largest Ministry of Health operational district in Kampong Speu Province and is the site of the Kampong Speu District Referral hospital, a hospital of approximately 100 beds with 130 staff of medical doctors, nurses and lab technicians. At this hospital, DENV fever is often clinically diagnosed (or sometimes confirmed by a SD Bioline Dengue Duo NS1/IgG/IgM rapid test when available) and reported to the National Dengue Control Programme, but the hospital does not serve as one of the five RT-PCR-confirmed dengue sentinel surveillance sites. Given its proximity to Phnom Penh, samples will be transported to the CNM laboratory for RT-PCR processing.

Four months prior to study start, a community engagement team (including the dengue provincial supervisor) was established and briefed on the overall objectives of the study along with the provincial clinical staff, including three pediatric specialist physicians who see patients in both the private and public sector clinics in Chbar Mon. The community engagement team then met with multiple village chiefs and their villagers across the three easternmost subcommunes of Chbar Mon for information sessions regarding the study. The community engagement team, in conjunction with the village chiefs and local monks, developed a recruitment schedule based on the availability of various community pagodas across Chbar Mon town. An estimated 97% of Cambodians are Buddhist and there are no other major enclaves of other religious groups in Chbar Mon, meaning that the use of pagodas is appropriate to gather a representative sample [28]. To enroll a wide variety of ages and villages within the Chbar Mon town, a different pagoda is designated for recruitment each week during the three-month enrollment period. Cohort enrollment will be structured for four age quadrants: 2–3 years of age, 4–5 years of age, 6–7 years of age, 8–9 years of age with the goal of approximately 192 children in each.

### Sample size calculation

There are no published seroprevalence studies exclusively for Cambodian children, but multiple pediatric seroprevalence studies are reported from neighboring Thailand and Malaysia [29–31]. The most recent incidence studies in Cambodia suggest that the most conservative estimate, or the lowest incidence rate, of acute dengue infection in Cambodia as approximately 14 per 1000 person-seasons (range 13.7–58 per 1000 person-seasons in neighboring province to Kampong Speu) [24].

It is difficult to make presumptions regarding the exposed mosquito saliva protein (MSP+) and unexposed mosquito saliva protein (MSP-) populations as the only available retrospective study data from Thailand suggested that seroconversion occurs to major *Ae. aegypti* proteins 0–25% of the time in non-infected hospitalized children *versus* 0–75% in DENV-infected hospitalized children [19]. Therefore, the sample size was primarily determined by the number necessary for DENV seroprevalence. With a sample size of 701 accounting for 10% dropout to yield a total goal enrollment of 771 participants, a simple random sample of a binary response (infected or not) with an exact 95% confidence interval on the true proportion will have an average length of less than or equal to 0.075 regardless of the true rate.

### Study objectives and endpoints

Table 1 provides a list of the primary, secondary and exploratory outcomes of the study and their associated endpoints and justifications.

### Clinical and laboratory procedures

At screening and enrollment, children and their guardians undergo informed consent and child identify verification (by village chief if child unable to verbalize the name of their guardian). Baseline demographics are collected, including: sex, age, residential address, dengue risk factors (e.g. number of domestic water containers at household, larvicide use), bednet use, past medical history, drug allergies, and any current or previous (within past month) use of over-the-counter, prescription, or traditional remedies. Participants undergo a targeted exam that includes vital signs, height and weight.

At baseline, trained phlebotomists collect 8 ml of peripheral blood in a heparin tube for peripheral blood mononuclear cell processing for future cellular assays and 6 ml in a serum separator tube (SST) for detection of dengue-specific antibodies using the PanBio Indirect IgG ELISA kit. For the serum samples that are found to be positive for DENV IgG, a plaque reduction neutralization test (PRNT) will be performed in order to determine the antibody titer to each of the four DENV serotypes. This will establish a baseline profile for each participant.

At semi-annual follow-ups, participants will undergo venipuncture of 8 ml in a serum separator tube for follow-up dengue antibody assessment *via* ELISA, and for those who have seroconverted to positive since baseline, follow-up PRNT assays will be done. At the first semi-annual follow-up, additional whole blood of up to approximately 2 ml will be taken for alpha-thalassemia typing and point-of-care hemoglobin testing. At the final visit of the study, 8 ml of whole blood in a heparin tube will again be taken for PBMC processing for future cellular assays.

Sick febrile visits may occur at any point during the three years. Upon enrollment, participants are given a thermometer to monitor their temperatures at home, call their Community Engagement liaison if they have a fever, and go to the Pediatrics ward at the referral hospital and present their study identification card to undergo vital signs, a dengue-specific sick questionnaire, targeted exam, and rapid diagnostic dengue test. Per Cambodian National Dengue Treatment Guidelines, all children with symptomatic dengue infection are hospitalized. If a patient is found to screen positive for dengue using the SD Bioline Dengue Duo kit, the participant undergoes the following: (i) Day 0 collection for RT-PCR confirmation of arboviral diagnosis, sera for *Ae. aegypti* SGH ELISA and dengue IgM dengue IgM/IgG ELISA, and PBMCs; (ii) Day 7 collection of 5 ml in SST for paired sera between acute and convalescent samples to detect IgM seroconversion or 4-fold increase in total anti-DENV antibodies as measured by ELISA; and (iii) Day 28 collection for 5 ml in SST for sera and 8 ml in heparinized tube for PBMCs for cellular assays. Complete blood counts are already performed as part of the hospitalization per national treatment guidelines, and this information is captured in the patient’s clinical research form (CRF). If the result of the dengue rapid test is negative, the physician will manage the participant as a non-dengue case and advise them accordingly. If the doctor still strongly suspects dengue despite negative rapid testing, or he/she suspects CHIKV or ZIKV infection, the study physician may request collection of 1 ml of whole blood in an EDTA tube to undergo Fast Track Diagnostics® RT-PCR multiplex testing for the viruses in the central CNM-NIH laboratory in Phnom Penh. Of note, no additional phlebotomy for research purposes at sick visits will be performed if a child just underwent venipuncture at a scheduled visit within the preceding 14 days.

### Data collection, monitoring, and storage of research samples

Upon enrollment in the protocol, all subjects will be assigned a unique identifying combination code of seven numbers (e.g. 100-0001). The identifier will serve to link this identifying code with subject name. CRFs, databases, and biological samples including DNA will be coded with unique identifying numbers and will not contain subject names. Study personnel involved in data acquisition, entry, and analysis will therefore not have access to names, assuring the privacy and confidentiality of subjects.

REDCap (Research Electronic Data Capture) serves as the core data collection component of the data management system hosted at NIAID. REDCap is a secure, web-based application designed to support data capture for research studies, providing: (i) an intuitive interface for validated data entry; (ii) audit trails for tracking data manipulation and export procedures; (iii) automated export procedures for seamless data downloads to common statistical packages; and (iv) procedures for importing data from external sources [32]. Standard CRFs containing clinical and laboratory data will be completed on iPads using the REDCap that will be locked with a code and uploaded to password-protected servers. Relevant subject data will be entered into a hybrid system (REDCap plus Datafax©). Electronically captured data will be checked by study investigators and verified by a data manager. All electronic data will be stored on a secure server.

Following written standard operating procedures (SOPs), external study monitors will verify that the clinical trial is conducted, data are generated, and biological specimens are collected, documented (recorded), and reported in compliance with the protocol, good clinical practices, and applicable regulatory requirements.

The PI will retain all study records for at least 5 years in compliance with institutional, institutional review board (IRB), state, and federal medical records retention requirements, whichever is longest. Data captured electronically *via* tablets will be backed up nightly to the site’s central server and transmitted on a weekly basis. All data will be archived at the end of the study and retained for a period of time consistent with IRB requirements.

All human biological samples will be received, processed, aliquoted, and stored at the central CNM laboratory in Phnom Penh according to standard laboratory procedures.

A geographical information system (GIS) was set up at the beginning of the study, following GPS training of survey and community engagement team members. All data are geographically referenced, with geographic coordinates (latitude, longitude and elevation) collected for all recruitment locations (participant pagodas), mosquito catch sites, and participant houses.

### Statistical analysis

The primary endpoints will be defined as:

(i) Seropositivity to DENV1-4 per indirect IgG ELISA (with plaque reduction neutralization assays performed as necessary to confirm inapparent dengue infections)
(ii) Seropositivity to *Ae. aegypti* salivary gland homogenate

Seroprevalence to each dengue serotype will be presented as a proportion for each timepoint. Unadjusted and adjusted logistic regression models will be performed evaluating the relationship between time and seroprevalence, with adjustments for age, year or season of collection, and socio-economic conditions (e.g. type of housing). Further, the seroconversion rate and change in the immune status will be determined by participants and separated by age group during the study period. Quasi-Poisson regression models will be fit to evaluate the relationship of age, year, socio-economic conditions (e.g. type of housing) to the primary endpoints of dengue infection or *Ae. aegypti* salivary gland homogenate seropositivity and to estimate prevalence ratios.

Because seroconversion to *Ae. aegypti* salivary gland homogenate is measured repeatedly on subjects over three years during wet and dry season, a single subject may be negative for seroconversion at one time, and positive at a later time. This will allow us to perform our secondary objective to evaluate the kinetics of the antibody response. Given these changing values, seroprevalence to *Ae. aegypti* salivary gland homogenate will be presented as proportion representing point prevalence for each season in each year of the study.

We will perform regression models (logistic for dichotomy predictors, quasi-Poisson models for incidence rates, linear models or mixed models for continuous predictors), to investigate the effect of environmental and socio-demographic conditions on the prevalence of *Aedes* salivary protein seroconversion (e.g. age, type of housing, number of domestic water containers) and season-specific data such a humidity, temperature and rainfall. All data will be geographically referenced. To evaluate spatial or temporal aggregation of salivary gland seroconversion, space-time scan statistics will be used. Exploratory spatial analyses will look for statistical clustering of disease, of IgG response and of mosquito abundance using both global and local indicators of spatial autocorrelation [33]. Smoothed heat maps will be created using QGIS software (https://www.qgis.org/en/site/). Spatial data will then be used to estimate risk factors for strongly positive antibody response to *Ae. aegypti* saliva and to risk of disease using generalized linear regression or generalized additive models (quasi-Poisson and logistic regression models), where appropriate.

The difference between groups in continuous secondary/exploratory outcomes will be tested by nonparametric Wilcoxon rank sum test, or if inferences on differences in means are desired, using a 2-sample t-test [34]. To test for differences among more than two groups, either a Kruskal-Wallis rank test (for nonparametric inferences) or a one-way ANOVA (for inferences on the means) will be used to test for overall differences. An appropriate data transformation (e.g. log_10_-transformation) may be applied to make inferences on ratios of geometrics means or to better satisfy assumptions of symmetry and homoscedasticity (similar variance).

Exploratory analyses for the relationship of *Ae. aegypti* saliva seroconversion with disease development will model the rate of incidence of composite *Aedes*-transmitted arboviral disease (as defined above) using a quasi-Poisson model. The primary interest will be in the relative risk (RR) of infection comparing at risk subjects with seroconversion to *Ae. aegypti* salivary protein to those without seroconversion at that time. Exposed will be defined as those with seroconversion to specific *Ae. aegypti* salivary protein while unexposed will be those without reactivity at baseline and each follow-up time point. For this analysis, seroconversion will be defined as a binary variable (present/absent) based on the level of antibody to *Ae. aegypti* salivary gland homogenate (positivity is generally considered two standard deviations above the mean optical density value for negative controls as determined by an ELISA assay with further adjustment as needed to account for cross-reactivity to *Anopheles* or *Culex* saliva).

To compare the rates in secondary/exploratory outcomes for significant differences in arboviral infection between participants with or without evidence of salivary protein exposures or to examine other groups such as alpha thalassemia variants, we will use quasi-Poisson models and central Fisher’s exact tests. As previously stated, secondary and exploratory analyses will evaluate ELISA values as background (negative), low, medium and high quartiles, based on range and as a continuous variable. From the subset of samples with the highest positive values from both primary and secondary analyses, Western blot assays will be used to identify the most immunodominant protein candidates for functional characterization and future studies on epitope identification and characterization. Western blot assays will also be run against *Culex* and *Anopheles* saliva to account for cross-reactive immunogenicity to mosquito saliva in general.

### Mosquito surveillance

In pupal surveys of *Aedes* mosquitos in Cambodia, 95.5% were *Ae*. *aegypti*, and the remainder were *Ae. albopictus*. This proportion was similar in both urban (94.7%) and rural (96.1%) areas and provides the basis for the observation that dengue in Cambodia is primarily transmitted by *Ae. aegypti* mosquitos [23]. There is no information available for *Aedes* biting rates in Kampong Speu or in Cambodia in general. In this part of Kampong Speu Province (Chbar Mon town), there is essentially no malaria transmission as infected *Anopheles* vectors are limited to the forested hills in the westernmost part of the province (in the Aoral region), approximately a 1–2 h drive by car [35]. *Culex* mosquitos are abundant throughout rural, peri-urban and urban areas of Cambodia and are responsible for transmitting JEV, predominantly in rural areas [36].

Mosquito sampling and house surveys will be used to assess temporal trends in mosquito abundance (by species), absence/presence of immature *Aedes* in houses and other house-related factors (building material, number of occupants etc.).

A polygon was drawn around the target study area, excluding subareas without housing (including forests, farms, rivers, highways and wetlands). A grid with 100 × 100 m cells was then imposed on the target area polygon. Two random points per cell were generated using the random point generator function in Quantum Geographical Information Systems (QGIS) (version 2.18.12). GPS units (Garmin etrex 20) were then used to located houses selected through random point generation within each grid cell. Surveyors first attempted the first random point and then defaulted to the second if the first house chose not to participate. When points did not fall on houses, the first house to the east was selected.

Approximately 800 houses are surveyed across the community recruitment zone and will continue to be surveyed each rainy season for documentation of immature *Aedes* species entomological indices as follows: House Index defined as larvae present per 100 houses; Container Index defined as proportion of containers positive for immature *Aedes*; and Bretau Index defined as number of positive containers per 100 houses inspected. These inspections are performed approximately monthly for one to three months surrounding blood sampling during transmission seasons, typically from June to September.

A central contiguous subarea within the total target area was selected for mosquito trapping. Grid cell centroids were chosen as locations for mosquito traps (roughly 100 m apart, based on *Aedes* flight range) [37]. The site was purposely chosen because of its central location, diversity of ecotypes (an urban area with edges on farms, river and forest), and because historically, community members from this area have had higher numbers of clinical dengue infections. GPS units were then used to select the nearest possible location for setting traps, defaulting to due east of the centroid when necessary. The gravid mosquito trap area covers 0.88 km^2^ of the central township. Collections of adult *Aedes* baited with hay infusions will occur weekly for approximately three months during transmission season but may be adjusted per yield of mosquitos. Community volunteers are trained to maintain and empty *Aedes* traps once per week. Mosquitos are identified by microscopy then stored with desiccant to be brought to the main Phnom Penh laboratory for storage and future molecular analyses.

## Discussion

Cambodia is rapidly changing and its urban population is projected to double in the next decade [21]. While dengue is transmitted in both rural and urban areas of Cambodia, an increase in urban and peri-urban slum-dwelling residents, paired with poor sanitation practices and flooding, raises the risk for arboviral disease outbreaks [22, 23]. In order to allocate scarce resources, the Ministry of Health needs access to high-quality data that can provide guidance regarding dengue epidemiology of both inapparent and symptomatic infection, individual risk factors such as water container numbers and larvicide use, characterization of the *Aedes* vector and its abundance in terms of traditional *Stegomyia* indices as well as anti-saliva antibodies that describe direct human-vector contact. Access to these baseline cohort data may aid in national decisions regarding effectiveness and implementation of vector control measures, epidemic forecasting, and/or arboviral disease risk assessments in the future.

In addition to providing important public health information on dengue and its mosquito vector, the study aims will contribute to the epidemiologic groundwork necessary to elucidate the relationship between arboviral disease development and anti-*Ae. aegypti* mosquito salivary peptide antibodies. The sialotranscriptome of *Ae. aegypti* has been characterized [38], and some immunomodulatory proteins have been identified. One 34-kDa salivary protein of unknown function was shown to: (i) suppress antimicrobial peptide secretion by keratinocytes; and (ii) inhibit type 1 IFN expression as well as its regulatory factors [39]. Another salivary protein, CLIPA3 protease, promotes DENV replication and dissemination *via* cleavage of extracellular matrix proteins, liquefying the dermal layer to theoretically permit interaction between virions and local immune cells that will subsequently migrate to draining lymph nodes to enhance DENV infectivity [10]. Recently, a 15-kDa salivary protein called LTRIN augmented ZIKV pathogenesis in *Ifnar -/-* mice by interfering with lymphotoxin receptor signaling, a communication link between the lymph nodes and surrounding stromal environment [9]. However, many mosquito salivary peptides remain unidentified and functionally uncharacterized.

Retrospective studies of human sera have demonstrated the usefulness of anti-*Aedes* salivary gland extract as: (i) a marker of *Aedes* exposure; and (ii) a general corollary of dengue transmission potential [17–19, 40, 41]. In studies in Colombia and France, it has been shown that anti-*Aedes* saliva antibodies can last at least 40 days and possibly up to 4 months [18, 42]. In Colombia, retrospective analysis of febrile patient sera demonstrated that IgG antibodies to whole *Ae. aegypti* salivary gland extract were significantly higher in DENV-viremic patients compared to non-infected controls, serving as a marker of vector exposure [18]. Studies have shown that IgG antibodies to SGE are more specific than IgM antibodies with less cross-reactivity to other mosquito vector species like *Culex* spp., but overall, *Culex* and *Aedes* appear to have species-specific saliva reactivity in terms of human antibody response given that they diverged over 55 million years ago [17, 42]. There is little cross-reactivity in the *Anopheles* genera that diverged more than 150 million years ago from *Aedes* [43]. While these studies on markers of exposure laid the groundwork for anti-*Aedes* antibody identification and assay development, they have yet to contribute to our understanding of saliva-mediated pathogenesis of arboviral disease in humans.

There is only one retrospective study published from Thailand evaluating the correlation of MSP antibodies to arboviral disease severity [19]. In this serosurvey of 101 paired samples, hospitalized Thai children with acute primary or secondary dengue infection demonstrated higher total IgG antibodies to total *Ae. aegypti* salivary gland extract compared to uninfected hospitalized controls, suggesting higher exposure to the vector correlated to higher risk of infection. However, of the three immunogenic saliva *Ae. aegypti* proteins identified in the study, there was no uniform expression pattern of antibody response magnitude to correlate to disease severity [19]. The lack of baseline samples and the cross-sectional retrospective nature of the study limited its conclusions. There are no published prospective studies of human antibody response to mosquito saliva in regards to arboviral disease development or pathogenesis.

Identification and functional characterization of mosquito-borne salivary determinants of disease could lead to novel approaches for the control and prevention of mosquito-borne disease. Prospective longitudinal cohort studies of leishmaniasis in Brazil and Mali have linked certain sand fly salivary protein seroconversion to the risk of disease development, and candidate salivary antigens have moved forward into pre-clinical development to be paired with pathogen-specific vaccines [44–46]. Hence, identifying immunodominant *Aedes* salivary proteins in the future *via* well-characterized prospective clinical cohorts will be the initial step to understand how mosquito saliva drives arboviral disease development in human patients.

Regarding other factors that may influence arboviral disease outcome, the alpha globin loci harbor a wide range of DNA sequence and structural variants in Cambodian people [47]. The most common are the -α^3.7^ deletion (16% allele frequency) and the Constant Spring mutation (αα^CS^, 2.3% allele frequency) found in a survey conducted in northwest Cambodia. The diversity and frequency of alpha globin gene variants in Phnom Penh and surrounding provinces are unknown. These alpha globin variants are functional and in homozygous form can cause anemia; however, little is known about the impact of alpha globin gene variants on dengue virus susceptibility or severity. Erythroid progenitors may be among the potential target cells of dengue virus infection [48], and erythroid precursors from patients with alpha thalassemia trait may be less capable of sustaining an dengue virus infection: alpha thalassemia trait cells had lower rates of infection in culture and higher rates of apoptosis [49]. Among patients on the more severe spectrum of alpha thalassemia, with hemoglobin H disease, the clinical course of dengue fever was different: patients tended to present with severe anemia rather than with hemoconcentration [50]. This interesting yet incomplete set of observations implies that dengue virus may infect erythroid progenitors and the disease progression may be modified by a patient’s alpha globin genotype. We propose collecting study participant DNA for the sole purposes of determining the alpha globin genotype, including alpha globin genotype in the models describing disease susceptibility and severity.

While one strength of the study is its prospective design to limit selection bias, the study is not powered to define acute dengue incidence, which would number children in the thousands. Therefore, in correlating with mosquito saliva antibody reactivity, the endpoints include both inapparent and symptomatic dengue cases. Further, the study is limited to children aged 2–9 years of age as this is the age strata at greatest risk of dengue. However, neighboring Thailand and Malaysia have recently witnessed a shift in age such that young adults (aged 16–25 years) are now at greatest risk of dengue infection [29, 31]. This study will be unable to comment on whether Cambodia is undergoing a similar demographic shift. Given the presence of multiple circulating flaviviruses in the region and an ongoing JEV vaccination campaign [51], it is possible that cross-reactivity between flaviviruses will cause false positivity on the dengue antibody ELISA assays. Dengue PRNT assays will be performed in order minimize the problem and will provide an important epidemiological assessment of asymptomatic or unreported dengue infections. Lastly, given the limitations of translating preclinical findings in animal models with controlled and known biting exposure to real-life human populations, the observed MSP reactivity patterns in this cohort study may not reflect actual human-mosquito contact as previous studies have shown some heterogeneity with regard to disease outcomes [17, 19, 52]. The frequency of exposure to mosquito bites of the cohort participants during the study is unmonitored and it may be a factor in influencing differences in MSP reactivity and dengue infection severity. For these reasons, well-characterized sera controls will be used in the ELISA assays as well as incorporating extensive demographic data, behavioral activities and disease incidence of apparent and inapparent dengue infections into statistical models.

Our long-term aim is to translate the aforementioned preliminary findings in animal models and retrospective cross-sectional human studies to better understand the immune response to *Aedes* mosquito saliva and its subsequent relationship to *Aedes*-transmitted arboviral disease development. To do so, it is necessary to lay the epidemiological foundation in order to rigorously address these questions. Our primary objective in this study is to establish a prospective cohort study to determine the seroprevalence of dengue and seropositivity to *Aedes* saliva in the Cambodian pediatric population. We hypothesize that the burden of dengue and *Aedes* exposure is great enough to support future studies to examine the complex human immune response to vector saliva in the setting of arboviral disease development. This has yet to be done in the Cambodian population and will be a critical first step. Future studies may be able to identify immunodominant *Aedes* salivary antigens that can serve as adjuvants or principal components in vaccines against the mosquito-borne viruses.
